# A Vegan Diet Is Associated with a Significant Reduction in Dietary Acid Load: Post Hoc Analysis of a Randomized Controlled Trial in Healthy Individuals

**DOI:** 10.3390/ijerph18199998

**Published:** 2021-09-23

**Authors:** Alexander Müller, Amy Marisa Zimmermann-Klemd, Ann-Kathrin Lederer, Luciana Hannibal, Stefanie Kowarschik, Roman Huber, Maximilian Andreas Storz

**Affiliations:** 1 Centre for Complementary Medicine, Department of Internal Medicine II, Faculty of Medicine, University of Freiburg, 79106 Freiburg, Germany; alexander.mueller@uniklinik-freiburg.de (A.M.); amy.klemd@uniklinik-freiburg.de (A.M.Z.-K.); ann-kathrin.lederer@uniklinik-freiburg.de (A.-K.L.); stefanie.kowarschik@uniklinik-freiburg.de (S.K.); roman.huber@uniklinik-freiburg.de (R.H.); 2 Medical Center, Laboratory of Clinical Biochemistry and Metabolism, Department of General Pediatrics, Adolescent Medicine and Neonatology, Faculty of Medicine, University of Freiburg, 79106 Freiburg, Germany; luciana.hannibal@uniklinik-freiburg.de

**Keywords:** vegan, plant-based, vegetarian, nutrition, dietary acid load, potential renal acid load, net endogenous acid production, diet, meat, health

## Abstract

The composition of diet strongly affects acid–base homeostasis. Western diets abundant in acidogenic foods (meat and cheese) and deficient in alkalizing foods (fruits and vegetables) increase dietary acid load (DAL). A high DAL has been associated with numerous health repercussions, including cardiovascular disease and type-2-diabetes. Plant-based diets have been associated with a lower DAL; however, the number of trials exploring this association is limited. This randomized-controlled trial sought to examine whether an isocaloric vegan diet lowers DAL as compared to a meat-rich diet. Forty-five omnivorous individuals were randomly assigned to a vegan diet (*n* = 23) or a meat-rich diet (*n* = 22) for 4 weeks. DAL was determined using potential renal acid load (PRAL) and net endogenous acid production (NEAP) scores at baseline and after 3 and 4 weeks, respectively. After 3 weeks, median PRAL (−23.57 (23.87)) and mean NEAP_R_ (12.85 ± 19.71) scores were significantly lower in the vegan group than in the meat-rich group (PRAL: 18.78 (21.04) and NEAP_R_: 60.93 ± 15.51, respectively). Effects were mediated by a lower phosphorus and protein intake in the vegan group. Our study suggests that a vegan diet is a potential means to reduce DAL, whereas a meat-rich diet substantially increases the DAL burden.

## 1. Introduction

It is now widely accepted that the composition of diet strongly affects acid–base homeostasis [1]. Dietary acid load (DAL) is a major determinant of systemic pH, metabolism and acid–base regulation [2]. A high DAL has been associated with insulin resistance [3], poor musculoskeletal health [4], an undesirable profile of cardiometabolic risk factors, incident chronic kidney disease [5,6] and poor mental health and sleep quality in women with type-2-diabetes [7].

Acidogenic foods include meat and meat products, cheese, fish, eggs and certain grains such as oats and processed wheat-based products [2,8]. Meat and meat products in particular are abundant in sulfur-containing amino acids (methionine, cysteine and homocysteine) [9]. Their oxidation generates sulfate, a non-metabolizable anion constituting a major determinant of the daily acid load [9,10]. The content of methionine and cysteine is 2- to 5-fold higher in eggs and meat than in certain grains and legumes [9], which, in turn, are considered alkalinizing foods. Chicken breast without skin and tuna contain 4.94 and 6.48 mg methionine/kcal, respectively, whereas pinto beans, lentils, corn and brown rice contain less than 1 mg methionine/kcal [11].

Vegetables and fruits are abundant in potassium salts of metabolizable organic anions (mainly malate and citrate), which undergo combustion in the body to yield bicarbonate and consume hydrogen ions when metabolized, thus having an alkalinizing effect [9,10,12].

The difference between these alkaline and acid products yields the dietary acid load [10,12]. Two scores are commonly used to estimate DAL in clinical and epidemiological trials [13], namely the potential renal acid load (PRAL) score, and the net endogenous acid production (NEAP) score. A positive PRAL value reflects acid-forming potential, whereas a negative PRAL value reflects alkaline-forming potential [14,15].

Some trials have suggested that plant-based diets (and vegan diets in particular) are linked to a lower DAL [10], yet the number of studies exploring this association is limited [2]. Most studies in that particular area of research had a descriptive cross-sectional design and did not evaluate the effects of dietary modifications [16,17,18].

A very recently published clinical trial demonstrated that a dietary modification from a Western diet toward a low-fat vegan diet significantly reduced DAL [2]. However, participants allocated to the vegan intervention group in this trial also experienced weight loss and had a significantly reduced daily energy intake, potentially suggesting reduced food intake. Thus, it remains uncertain whether the reduction in DAL is attributable to a reduced energy intake or to a modification of the dietary composition.

The present study sought to investigate this problem. The major aims were two-fold: (1) to investigate whether a short-term isocaloric vegan dietary intervention reduces DAL in healthy individuals; and (2) to contrast the results to the effects of a meat-rich diet.

## 2. Materials and Methods

### 2.1. Study Design

The present study is a post hoc analysis of a randomized controlled trial we reported earlier [19]. The study design has been described elsewhere in detail [19,20]. In brief, we initially performed a monocentric, randomized-controlled pilot trial with parallel group design at the Center for Complementary Medicine at Freiburg University, Germany. Between April and June 2017, healthy, normal weight individuals (Body Mass Index (BMI) between 18.5 and 30 kg/m^2^), aged 18 to 60 years, without clinically relevant allergies were enrolled. Prior to the study, all participants consumed an omnivorous diet.

Eating disorders, participation in another clinical trial and blood donations within 4 weeks prior to enrollment yielded reasons for ineligibility. Additional exclusion criteria included abuse of drugs, nicotine or alcohol as well as a regular intake of medication. Individuals consuming a plant-based diet prior to the study were not considered eligible. Obese individuals and individuals aged 60 years or older were considered ineligible. Both factors were seen as potential confounders that may (negatively) affect immunological and metabolic parameters (primary study aim, see statistical analysis) [21,22]. Participants had to be proficient in German and were asked to complete a weekly nutritional protocol (“Freiburger Ernährungsprotokoll”) [23], which was mandatory for participation. All participants received extensive training prior to the study on how to handle the protocols. Protocols were analyzed using NutriGuide^®^ software (Version 4.7, Nutri-Science GmbH, Hausach, Germany). Based on Willett’s criteria, we considered only (complete) nutritional protocols with a plausible energy intake (≥800 kcal/day) for the present study [24]. Participants who reported consuming fewer than 800 kcal/d were excluded from the analysis. Participants who provided nutritional protocols with more than one missing day per week were also excluded. As a consequence, the included study population slightly differs from Lederer et al. (2019) [19].

We used newspaper announcements and local bulletins for recruitment. Eligible individuals were invited for a personal interview to check eligibility criteria in detail. After signing written informed consent, participants entered a one week run-in-phase. For seven days, participants were asked to eat a balanced (mixed) omnivorous diet according to the recommendations of the German Nutrition Association (DGE) [25].

Afterwards, participants were randomly assigned to either a meat-rich (>150 g of meat per day; any meat of their choice) or a vegan diet (defined as excluding all animal products) for four weeks. Extensive training on the assigned diet was given to all participants. Participants were free to choose foods within their assigned diet, and no pre-cooked meals were provided. During the study, all participants had free-of-charge access to meat-rich or customized vegan meals at the restaurant of the University Hospital Freiburg. Some local restaurants also offered discounts for study participants. Finally, participants were requested to keep their caloric intake stable to avoid weight loss.

The ethical committee of the University Medical Center of Freiburg, Germany (EK Freiburg 38/17) approved the trial. We registered the trial at the German Clinical Trial register (DRKS00011963) before onset. The study was performed according to the principles of the declaration of Helsinki and to the guidelines of ICH (International Conference on Harmonization) for good clinical practice (GCP). A third independent person created an electronically block-wise randomization list (block size 13; Python Software), and sealed envelopes were used for implementation.

### 2.2. Dietary Acid Load Calculations

In order to calculate dietary acid load, we used three widely established formulas which were introduced by Remer et al. and Frasetto et al. [14,26]. Potential renal acid load (PRAL) of diet was calculated as follows:

PRAL (mEq/day) = (0.49 × total protein (g/day)) + (0.037 × phosphorus (mg/day)) − (0.021 × potassium (mg/day)) − (0.026 × magnesium (mg/day)) − (0.013 × calcium (mg/day))(1)

This score includes intestinal absorption rates for the following micro- and macronutrients: potassium, phosphate, magnesium, calcium and protein. Moreover, it considers ionic dissociation and sulfur metabolism [14]. This method of calculation was validated against urinary renal net acid excretion and reliably estimates the acid load from diet [14]. Net endogenous acid production (NEAP) was calculated using two different formulas: NEAP_R_ (as proposed by Remer and Manz [14,27]), and NEAP_F_ (as proposed by Frasetto et al. [26]).

Remer et al. estimated net endogenous acid production from average intestinal absorption rates of ingested protein and additional minerals (PRAL-score) as well as anthropometry-based estimates for organic acid excretion (OA_est_) [14]:

Estimated NEAPR (mEq/d) = PRAL (mEq/d) + OAest (mEq/d)(2)

whereby OA_est_ (mEq/d) was calculated as follows:

Individual body surface area × 41/1.73(3)

Body surface area was calculated according to the formula of Du Bois and Du Bois as follows:

Body surface area (m^2^) = (0.007184 × height (cm)^0.725^ × weight (kg)^0.425^) (4)

Frasetto et al. estimated a diet’s net acid load (NEAP_F_) from the dietary content of potassium and protein [28]:

Estimated NEAP_F_ (mEq/d) = (54.4 × protein (g/d)/potassium (mEq/d)) − 10.2 (5)

Given the fact that each of the aforementioned algorithms has its drawbacks and merits [16], we applied both models (NEAP_F_ and NEAP_R_) and examined how they alter NEAP in vegan and meat-rich diets.

### 2.3. Statistical Analysis

The present study is a post hoc analysis of a randomized controlled trial. The aim of the initial pilot trial was to broadly map metabolomic, microbial and immunological changes in healthy participants after adopting a vegan diet compared to a meat-rich diet. Sample size calculation for the initial RCT was planned for three different immunological main outcome parameters considering a statistical power of 80% and a hypothesized large effect size. An a priori sample size calculation revealed that 48 participants (24 for each diet) would be required to detect a statistical difference of *p* < 0.05 between the groups. Four additional participants were included to reserve for drop-outs. Forty-five participants (23 in the vegan group, 22 in the meat-rich group), characterized by complete and plausible nutritional protocols, were included in this secondary data analysis. Potential renal acid load and net endogenous acid production were exploratory analyses. Data were entered blinded for diet assignment in a preformed table.

Statistical analysis was performed using R (R version 4.1.0, The R Foundation for Statistical Computing) [29]. Extreme outliers in the data were removed if they fell above the 75th or below the 25th percentile by a factor of 3 times the interquartile range. As most of the data were not normally distributed (Shapiro–Wilk test, at significant level set to α > 0.05), the macro- and micronutrient intake data as well as the DAL scores data were fitted to a generalized linear mixed model (GLMM) with distributions fitted specifically for each nutrient intake and score dataset (total energy and magnesium intake followed a Gamma distribution; protein, potassium, calcium and phosphorus intake followed a Log-normal distribution; PRAL, NEAPR and NEAPF scores followed a Normal distribution). Fixed effects were defined as *Group* (two levels: Vegan diet and Meat-rich diet) and *Time* (three levels: Run-in, Week 3 and Week 4). *Subjects* was used as random effect with a random intercept. An analysis of deviance based on the mixed linear model was then implemented to investigate any interaction effects. If an interaction effect was significant (significant level set to α = 0.05), follow-up post hoc tests with Tukey adjustment were implemented.

## 3. Results

We screened 150 interested individuals for eligibility by phone call; 103 individuals were invited to a personal interview, and 61 participants started the run-in phase. From these, eight had to be excluded before randomization due to acute illness or (late) withdrawal of consent. Fifty-three participants completed the run-in-phase and started the intervention period. Twenty-seven participants were allocated to the meat-rich diet, and 26 were allocated to the vegan diet. All 53 participants completed the study as per protocol.

Eight participants provided nutritional protocols that did not meet our inclusion criteria. As such, only 45 participants were included in the final analysis (*n* = 23 in the vegan group and *n* = 22 in the meat-rich diet group). Table 1 displays demographic and anthropometric baseline data of all participants.

Mean age of all participants was 31.28 years. With regard to age, weight, height and BMI, we observed no significant intergroup differences at baseline (Table 1).

Table 2 displays the results from the nutritional protocol analysis of the first week (“run-in-phase”), where all participants were assigned to the same balanced (mixed) omnivorous diet according to the recommendations of the German Nutrition Association [25]. Results from week 3 and week 4 (after group assignment) are shown as well.

We observed no significant intergroup differences during the run-in-phase with regard to protein intake, potassium intake, magnesium intake, calcium intake and phosphorus intake (Table 2). Median total energy intake was slightly higher in the second group (2220.5 (955.75) kcal/d) as compared to the first group (2085 (770) kcal/d); however, this difference was not statistically significant (*p* = 0.930).

After three weeks into the trial, median protein intake decreased substantially in those participants assigned to the vegan group. Protein intake fell from 81.66 (26.73) g/d to 61.28 (51.11) g/d. In comparison, median protein intake increased with a meat-rich diet (from 90.62 (42.31) g/d during the run-in-phase to 99.30 (42.76) g/d in week 3. The intergroup difference in protein intake was statistically significant (*p* ≤ 0.001), a phenomenon that we also observed in week 4 (Table 2).

**Figure 1:**
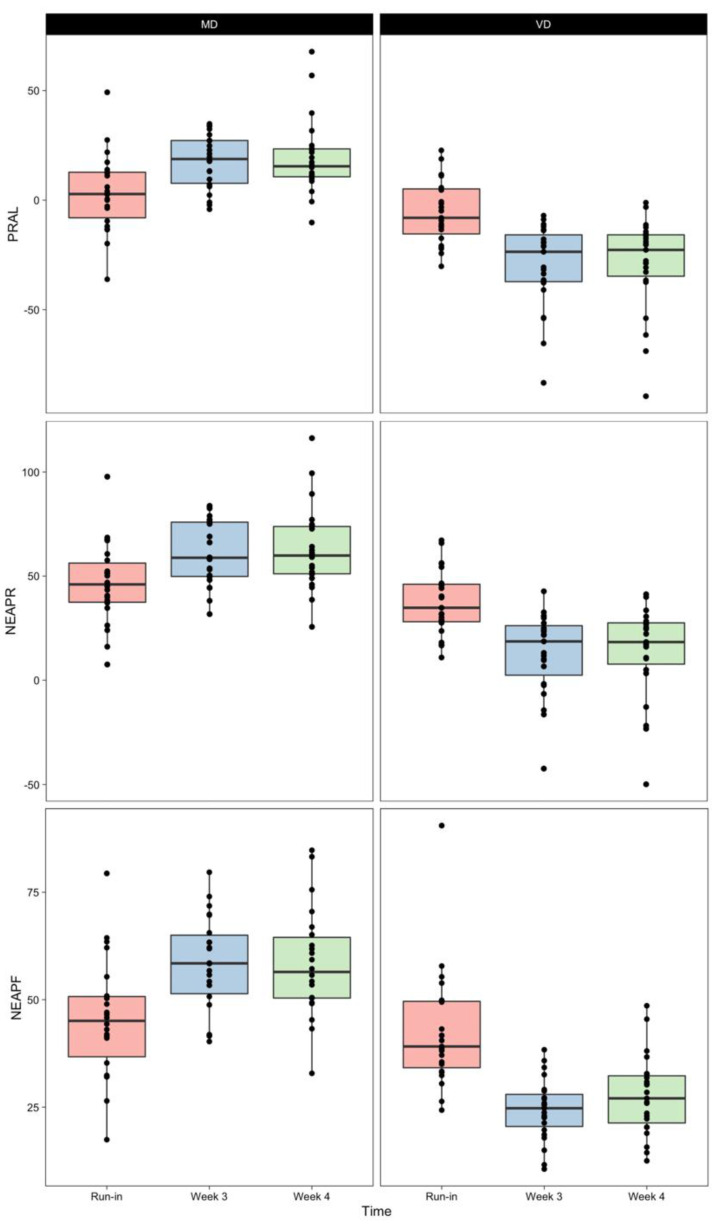
Box plots of DAL indexes PRAL, NEAP_R_ and NEAP_F_ calculated after the run-in phase (red), after 3 weeks of dietary intervention (blue) and after 4 weeks of dietary intervention (green) with either a meat-rich (MD) or a vegan diet (VD). Lower and upper hinges of the box plots delimit 25th and 75th percentiles. The middle line shows the median. Upper and lower whiskers extend from their respective hinges to the largest and lowest value, no further than 1.5 times the inter-quartile range, respectively.

Table 3 displays the mean/median DAL scores that we calculated based on the aforementioned formulas (see dietary acid load calculations). Indexes PRAL, NEAP_R_ and NEAP_F_ were comparable between the randomized groups after the 7-day run-in phase. Dietary intervention with meat-rich and vegan diets significantly modified PRAL, NEAP_R_ and NEAP_F_ after 3 and 4 weeks (Figure 1).

After the run-in-phase, mean PRAL values were lower (−5.26 ± 4.45) in the group that was later assigned a vegan diet than in the group that was later assigned a meat-rich diet (3.26 ± 17.91), yet this difference was not statistically significant (*p* = 0.492). Moreover, we observed no significant intergroup differences with regard to NEAP_R_ and NEAP_F_ values at baseline (Table 3).

After 3 weeks, all dietary acid load scores fell significantly in the vegan group (*p* < 0.001): mean PRAL-scores decreased by −24.608 mEq/d (run-in-phase vs. week 3, *p* ≤ 0.001); NEAP_R_ decreased by −24.607 mEq/d (run-in-phase vs. week 3, *p* ≤ 0.001). We also observed a comparable decline in median NEAP_F_ scores (−18.081 mEq/d; run-in-phase vs. week 3, *p* ≤ 0.001).

In contrast, our results demonstrate a substantial increase in DAL scores in the meat-rich diet group. Mean PRAL scores increased by 14.35 mEq/d (run-in-phase vs. week 3, *p* ≤ 0.001), and mean NEAP_R_ scores increased by 14.360 mEq/d (run-in-phase vs. week 3, *p* ≤ 0.001). A pronounced increase was also observed with mean NEAP_F_ scores (+12.63 mEq/d, run-in-phase vs. week 3, *p* ≤ 0.001). Results from week 4 yielded a comparable picture to week 3 with significant decreased in all three DAL scores in the vegan group (run-in-phase vs. week 4, *p* ≤ 0.001 for PRAL, NEAP_R_ and NEAP_F_)_._

Of note, average calorie intake remained relatively stable over the course of the study (Table 2), indicating good participant adherence to our study protocol (requesting participants to keep their total calorie intake constant) [19]. Participant weight did not change substantially during the course of the study. Participants in the vegan group had a median weight of 65 kg (10.35) after 4 weeks (vs. 64.9 (11.45) kg at baseline), whereas participants in the meat-rich group had a median weight of 68.1 (20.8) kg after the same period (vs. 67.95 (20.75) kg at baseline).

## 4. Discussion

Our results confirm the hypothesis that a short-term (isocaloric) vegan dietary intervention effectively reduces DAL in healthy individuals, whereas a meat-rich diet increases it. Median PRAL, NEAP_F_ and NEAP_R_ scores decreased significantly in the vegan intervention group (Table 3 and Figure 1). These findings are of paramount importance, as a high DAL has been associated with a series of health repercussions [8], including an increased risk for cardiovascular disease [30], type-2-diabetes [31], metabolic syndrome [32], chronic kidney disease [33] and an elevated lipid accumulation product [34].

Our results are consistent with the majority of intervention studies investigating the DAL-lowering effects of various plant-based diet patterns [2,10,18]. In a recently published trial, Kahleova et al. randomized 244 overweight adults to either an ad libitum low-fat vegan diet (LVFD) or a control diet [2]. The LFVD predominantly included grains, legumes, vegetables and fruits and was characterized by a targeted macronutrient distribution of ~75% of energy from carbohydrates, 15% protein and 10% fat. The control group was requested to avoid any dietary modifications. After 16 weeks, median PRAL and NEAP_F_ scores fell significantly in the vegan intervention group (−24.3 (−28 to −20.5) mEq/day and −25.1 (−29.1 to −21.1) mEq/day, respectively). In comparison, both scores remained almost identical in the control-group (PRAL: +0.4 (−3.6–4.5); NEAP_F_: −1.3 (−5.5–3.0).

A 2017 study by Cosgrove and Johnston compared the PRAL-lowering effects of three different vegan intervention patterns including a vegan diet for seven consecutive days (VEG7) and a vegan diet followed for two or three days over one week (VEG2+3) [10]. While only the VEG7 intervention significantly increased 24-h urine pH, PRAL scores fell significantly in all groups. Effects were more pronounced in those individuals following a vegan diet for 7 days (PRAL-scores dropped by 29.7 mEq/d, from 23.7 ± 16.7 to −6.0 ± 12.8) than in individuals who followed the vegan diet for only 2 or 3 days (PRAL-scores dropped by 12.8 mEq/d, from 18.1 ± 10.7 to 5.3 ± 11.4).

In both of the aforementioned studies, (strict) vegans yielded negative PRAL-values following the dietary intervention (−20.7 (−23.3 to −18.1) and −6.0 ± 12.8) [2,10]. It is noteworthy that there is also some evidence suggesting that a lacto–ovo-vegetarian diet (including dairy and eggs) may have PRAL-lowering effects (as compared to a non-vegetarian diet). Deriemaker et al. estimated DAL in lacto–ovo-vegetarians and found lower PRAL-scores in this group (−5.4 ± 12.4) as compared to non-vegetarians who exhibited positive PRAL values (10.3 ± 14.4) [18].

These studies indicate that the composition of a plant-based diet is of paramount importance to reduce the burden from DAL. Lacto–ovo-vegetarian diets include eggs, cheese and other dairy products, which are abundant in phosphorus and preservative phosphate (phosphoric acid, polyphosphates) [35]. Both are characterized by a high gastrointestinal absorption rate and therefore contribute to an elevated DAL [12]. Vegan diets, in contrast, restrict dairy products and replace them with plant-foods. These foods contain phosphorus in the form of phytate, which has a lower bioavailability and therefore no acidizing effects [2].

Another factor contributing to the different effects of a (strict) vegan diet and a lacto–ovo-vegetarian diet on DAL is the different dietary protein composition. Large epidemiological investigations revealed that lacto–ovo-vegetarian diets are usually higher in total protein than vegan diets [36]. In contrast, vegan diets include substantially more plant protein [37]. A prominent example is the French NutriNet-Santé Study, where vegetarians consumed, on average, 33.8 g of plant-protein per day, whereas vegans ate 46.5 g of plant-protein per day [37]. This translates into a significantly higher intake of fruits, legumes and vegetables, which generally have an alkalizing effect. These foods are also abundant in potassium, which releases (alkalizing) precursors of bases in the bloodstream [38].

Protein and phosphorus intake have been significantly associated with an increased acid load [39]. In our sample, the large DAL intergroup difference is most likely mediated by a lower protein and phosphorus intake in the vegan group, as compared to the meat-rich diet group (Table 2).

The present clinical trial has several strengths and limitations that warrant further investigation. Strengths include the randomized-controlled design of our trial and the inclusion of three different DAL scores. As opposed to many other clinical intervention studies, we also reported NEAP_R_, which is based on organic acid excretion (OA_est_) and the PRAL-score defined by Remer and colleagues [14]. The number of trials investigating dietary acid load following adoption of a plant-based diet is limited, and most studies have a purely descriptive cross-sectional design. DAL scores in the present studies are based on daily nutritional protocols following a dietary intervention in a randomized-controlled setting.

Weaknesses include the rather small sample size and the lack of a systematic “food intake pattern analysis”, which would have allowed additional insights into the PRAL-lowering effects of certain food groups. In addition to that, nutritional studies are often subject to (dietary) recall bias. Since we only included participants that provided plausible nutritional protocols at all three measure points, we had to exclude eight participants in total. These participants provided either incomplete protocols (*n* = 5) or reported consuming fewer than 800 kcal/d (*n* = 3). Food frequency questionnaires (FFQs) generally have intrinsic limitations (e.g., marked frequency of consumption and portion size may not represent usual intake of respondents) and require certain literacy and cognitive skills [40]. Food intake in vegan diets is best assessed with special (yet large) FFQs (e.g., [41]) that were not used in the present study for practical reasons. Finally, it is noteworthy that participants in both groups were instructed to keep caloric intake stable to maintain their weight. Vegan diets, however, are usually characterized by a reduced caloric density and a high nutrient density [42,43]. These features promote earlier satiety [44] and contribute to a lower total calorie intake. Several participants assigned to the vegan group in our study (occasionally) consumed acidifying grain-based snacks, processed wheat-products such as granola bars and sweets to reach the target of approximately 2000 kcal/d. Of note, some grains such as oats and processed wheat-based products are considered acidogenic foods [2,8]. Thus, it is conceivable that we might have (slightly) underestimated the DAL-lowering effect of diet in the vegan group. Finally, it is important to note that we only measured each participant’s weight at the beginning (baseline) and at the end of the intervention (after 4 weeks). During the intervention period itself, participants measured their weight at home. With regard to the missing values at week 3, we consistently used the participant’s baseline weight to calculate NEAP_R_. Although participants’ weights did not change substantially over the course of the study, we acknowledge that this procedure may introduce a small degree of inaccuracy to the estimations.

While our study provides insights in the field of plant-based nutrition and dietary acid load, we believe that additional (larger) trials are warranted to confirm the recent findings by others and us. Additional studies should ideally be supported by DAL-related biomarkers and include disease-related clinical endpoints. Finally, we believe that comparable studies in older populations would also be interesting, given that recent studies suggested a relatively higher production of NEAP in older people [45].

## 5. Conclusions

Our study adds to the evidence that a vegan diet results in a lower DAL burden as opposed to a meat-based diet. These findings are of high clinical relevance, as a high DAL has been associated with negative health outcomes. Future studies (supported by DAL-related biomarkers) are necessary to confirm our findings and should also compare the different plant-based dietary patterns (lacto–ovo-vegetarian, vegan, whole-food plant-based) in a randomized-controlled manner.

## Figures and Tables

**Table 1:** Participants’ baseline data. Normally distributed data are shown as mean ± standard deviation; not normally distributed data are shown as medians (interquartile range). Chi-Square Test was used to calculate *p*-values for gender.

Variable	Vegan Diet Group (*n* = 23)	Meat-Rich Diet Group (*n* = 22)	*p*-Value
Gender			0.098
Male	*n* = 6	*n* = 11	
Female	*n* = 17	*n* = 11	
Age (years)	30 (11.45)	26.5 (11.25)	0.241
Weight (kg)	68.73 ± 11.18	69.35 ± 13.30	0.864
Height (cm)	172.26 ± 9.20	173.45 ± 10.93	0.693
Body mass index (kg/m^2^)	22 (2.10)	23.00 (3.15)	0.937

**Table 2:** Macro- and micronutrient intake during the run-in-phase and after 3 and 4 weeks across the vegan and the meat-rich diet group: A comparison. Normally distributed data are shown as mean ± standard deviation; not normally distributed data are shown as medians (interquartile range). *p*-values from post hoc tests for which Group × Time interactions were significant, except indicated by †. Number of participants used for statistical analysis after outlier removal: (a) vegan diet group, *n* = 22, meat-rich diet group, *n* = 21; (b) vegan diet group, *n* = 21, meat-rich diet group, *n* = 20; (c) vegan diet group, *n* = 21, meat-rich diet group, *n* = 20; (d) vegan diet group, *n* = 23, meat-rich diet group, *n* = 20; (e) vegan diet group, *n* = 22, meat-rich diet group, *n* = 21; (f) vegan diet group, *n* = 21, meat-rich diet group, *n* = 20.

Variable	Vegan Diet Group (Total *n* = 23)	Meat-Rich Diet Group (Total *n* = 22)	*p*-Value
**Run-in-phase**			
Total Energy Intake (kcal/d) ^a^	2085.00 (770)	2220.50 (955.75)	0.930 ^“^^†”^
Protein intake (g/d) ^b^	81.66 (26.73)	90.62 (42.31)	0.974
Potassium intake (mg/d) ^c^	3613.74 (1027.26)	3495.24 (802.91)	0.998 ^“^^†”^
Magnesium intake (mg/d) ^d^	380.12 (143.95)	375.77 (151.93)	0.999
Calcium intake (mg/d) ^e^	862.40 (457.99)	876.11 (423.48)	0.989
Phosphorus intake (mg/d) ^f^	1238.74 (318.50)	1453.64 (485.57)	0.956
**Week 3**			
Total Energy Intake (kcal/d) ^a^	1811 (1274)	2083.5 (1034)	0.811 ^“^^†”^
Protein intake (g/d) ^b^	61.28 (51.11)	99.30 (42.76)	<0.001
Potassium intake (mg/d) ^c^	3936.49 (2841.44)	3048.58 (975.41)	0.486 ^“^^†”^
Magnesium intake (mg/d) ^d^	390.01 (321.39)	325.54 (178.19)	0.314
Calcium intake (mg/d) ^e^	478 (355.73)	871.12 (531.97)	0.059
Phosphorus intake (mg/d) ^f^	918.99 (756.64)	1375.69 (597.34)	0.096
**Week 4**			
Total Energy Intake (kcal/d) ^a^	2137.30 ± 866.952	2401.36 ± 868.155	0.895 ^“^^†”^
Protein intake (g/d) ^b^	67.10 ± 30.05	110.44 ± 39.72	<0.001
Potassium intake (mg/d) ^c^	3842.08 ± 1747.23	3444.42 ± 1102.35	0.990 ^“^^†”^
Magnesium intake (mg/d) ^d^	463.33 ± 216.77	384.33 ± 157.48	0.767
Calcium intake (mg/d) ^e^	515.42 (372.28)	917.38 (558.34)	0.003
Phosphorus intake (mg/d) ^f^	1122.06 ± 509.82	1595.65 ± 620.08	0.015

**Table 3:** DAL scores during the run-in-phase and at week 3 and 4 week across the vegan diet and the meat-rich diet group: A comparison. Normally distributed data are shown as mean ± standard deviation; not normally distributed data are shown as medians (interquartile range). *p*-values from post hoc tests for which Group × Time interactions were significant. Number of participants used for statistical analysis after outlier removal: (a) vegan diet group, *n* = 23, meat-rich diet group, *n* = 21; (b) vegan diet group, *n* = 23, meat-rich diet group, *n* = 22; (c) vegan diet group, *n* = 23, meat-rich diet group, *n* = 22.

Variable	Vegan Diet Group (Total *n* = 23)	Meat-Rich Diet Group (Total *n* = 22)	*p*-Value
**Run-in-phase**			
PRAL (mEq/day) ^a^	−5.26 ± 4.45	3.26 ± 17.91	0.492
NEAPR (mEq/day) ^b^	37.45 ± 15.73	46.57 ± 19.69	0.574
NEAPF (mEq/day) ^c^	39.11 (16.45)	45.07 (17.44)	0.944
**Week 3**			
PRAL (mEq/day) ^a^	−23.57 (23.87)	18.78 (21.04)	<0.001
NEAPR (mEq/day) ^b^	12.85 ± 19.71	60.93 ± 15.51	<0.001
NEAPF (mEq/day) ^c^	24.39 ± 7.1	58.32 ± 11.19	<0.001
**Week 4**			
PRAL (mEq/day) ^a^	−22.71 (21.25)	15.47 (13.51)	<0.001
NEAPR (mEq/day) ^b^	18.31 (22.83)	59.87 (23.91)	<0.001
NEAPF (mEq/day) ^c^	27.65 ± 9.1	58.29 ± 12.67	<0.001

## Data Availability

The data presented in this study will be made available upon reasonable request from the corresponding author.
